# A Serum Factor Induces Insulin-Independent Translocation of GLUT4 to the Cell Surface which Is Maintained in Insulin Resistance

**DOI:** 10.1371/journal.pone.0015560

**Published:** 2010-12-20

**Authors:** Marion Berenguer, Laurène Martinez, Sophie Giorgetti-Peraldi, Yannick Le Marchand-Brustel, Roland Govers

**Affiliations:** 1 INSERM, U895, Mediterranean Research Center for Molecular Medicine (C3M), Avenir Team 9, Nice, France; 2 INSERM, U895, Mediterranean Research Center for Molecular Medicine (C3M), Team 7, Nice, France; 3 University of Nice-Sophia-Antipolis, Faculty of Medicine, Signaling and pathologies (IFR50), Nice, France; University of Cambridge, United Kingdom

## Abstract

In response to insulin, glucose transporter GLUT4 translocates from intracellular compartments towards the plasma membrane where it enhances cellular glucose uptake. Here, we show that sera from various species contain a factor that dose-dependently induces GLUT4 translocation and glucose uptake in 3T3-L1 adipocytes, human adipocytes, myoblasts and myotubes. Notably, the effect of this factor on GLUT4 is fully maintained in insulin-resistant cells. Our studies demonstrate that the serum-induced increase in cell surface GLUT4 levels is not due to inhibition of its internalization and is not mediated by insulin, PDGF, IGF-1, or HGF. Similarly to insulin, serum also augments cell surface levels of GLUT1 and TfR. Remarkably, the acute effect of serum on GLUT4 is largely additive to that of insulin, while it also sensitizes the cells to insulin. In accordance with these findings, serum does not appear to activate the same repertoire of downstream signaling molecules that are implicated in insulin-induced GLUT4 translocation. We conclude that in addition to insulin, at least one other biological proteinaceous factor exists that contributes to GLUT4 regulation and still functions in insulin resistance. The challenge now is to identify this factor.

## Introduction

GLUT4 is the principle glucose transporter that is responsible for the insulin-induced uptake of glucose by muscle and adipose tissue after a meal. The main characteristic feature of GLUT4 is the absence of cell surface recycling in non-stimulated cells [1]. This implicates the presence of a highly efficient cellular mechanism that retains GLUT4 intracellularly. How this retention is organized and which molecules are implicated is currently unknown. It has been postulated that the non-endosomal GLUT4 pool plays a major role in intracellular retention [2]. Nevertheless, albeit to a lesser extent, endosomal GLUT4 has been demonstrated to be retained intracellularly and responsive to insulin [3], [4]. In accordance, GLUT1 and the transferrin receptor (TfR), both localized exclusively in endosomes, also translocate to the plasma membrane upon insulin stimulation [2], [5]. GLUT4 is regulated by insulin at various levels. Insulin signaling reduces GLUT4 retention allowing GLUT4 to move towards the cell periphery [6], increases its endosomal recycling [7], and enhances its docking and fusion with the plasma membrane [8]. On the other hand, GLUT4 internalization is hardly regulated by insulin, at least in adipocytes [4], [9]. Despite the fact that many players in insulin signaling and GLUT4 traffic are known, it remains elusive how these two pathways intercommunicate.

In insulin resistance, a condition related to type 2 diabetes, insulin no longer leads to an efficient translocation of GLUT4 towards the cell surface. Insulin resistance has been associated with a number of cellular phenomena which are likely to be linked. First, the level of reactive oxygen species (ROS) is increased under conditions of insulin resistance, while antioxidants are able to ameliorate insulin sensitivity and glucose uptake [10], [11]. Second, insulin resistance has been associated with a reduction in the phosphorylation (activity) of insulin signaling molecules [12], [13]. Third, increases in *O*-GlcNAc modification of proteins is linked to insulin resistance [14]. Notably, PKB, IRS-1, munc18c, as well as GLUT4 itself were found to be modified by O-GlcNAcylation [15], [16]. Fourth, in insulin resistance, expression levels of molecules implicated in insulin-induced GLUT4 translocation are decreased [17], [18]. Finally, insulin resistance has been associated with a change in the intracellular localization of GLUT4 [19], [20].

Taken together, multiple cellular mechanisms contribute to the development of insulin resistance and the associated reduction in GLUT4-mediated glucose uptake. It would be therapeutically relevant to discover novel ways to increase cell surface GLUT4 levels. Here, we present evidence for the existence of a putative factor in serum that displays an insulin-like effect regarding GLUT4 translocation and cellular glucose uptake. Remarkably, the effect of this factor on GLUT4 is additive to the action of insulin and is fully maintained in insulin resistance.

## Materials and Methods

### Materials

3T3-L1 preadipocytes were obtained from ATCC/LGC Standards (Teddington, UK). Plat-E cells were generously provided by Dr Toshio Kitamura (University of Tokyo, Japan). Bovine and human sera were from PAA (Pasching, Austria). Rabbit serum was withdrawn from New Zealand rabbits. Media and HEPES were from Invitrogen (Carlsbad, CA), insulin from Lilly (Suresnes, France), PDGF-BB, IGF-1, and HGF from PeproTech (Rocky Hill, NJ), and 2-[3H]deoxyglucose from PerkinElmer Life (Waltham, MA). Monoclonal anti-HA antibody was from Covance (Emeryville, CA), mouse IgG antibody from Sigma-Aldrich (St. Louis, MO), polyclonal GLUT4, GLUT1, and insulin receptor (IRβ) antibodies and HRP-conjugated secondary antibodies from Santa Cruz (Santa Cruz, CA), antibodies against PAS, ERK, phospho-ERK, Akt, phospho-Akt (T308 and S473), AMPK, phospho-AMPK, and phosphotyrosine from Cell Signaling Technology (Danvers, MA), anti-AS160 from AbCam (Cambridge, MA), and anti-phospho-AS160, anti-TfR and fluorescent antibodies from Invitrogen. IRS-1 antibody has been described before [21]. Polyclonal antibody against syntaxin 13 was generously provided by Dr Rytis Prekeris (University of Colorado, Denver, CO). Insulin ELISA kit was purchased from Spi-Bio (Montigny le Bretonneux, France) and inhibitors from Sigma (St. Louis, MO; wortmannin, LY294002, genistein), Calbiochem/Merck (Nottingham, UK; AG1024, Compound C, Akti 1/2, Ro 31-8220, U-73122, okadaic acid, U0126, rapamycin) and Alexis Biochemicals/Enzo Life Sciences (Plymouth Meeting, PA; herbimycin A, staurosporine). All chromatography materials were from Sigma. pBABE vector was kindly provided by Dr Hartmut Land (University of Rochester, Rochester, NY) and GLUT1 cDNA by Dr Mike Mueckler (Washington University, St. Louis, MO).

### Molecular Biology

The cDNAs encoding HA-GLUT4 (GLUT4 with an HA epitope tag in its first luminal domain) and HA-TfR (TfR with an HA tag at its C-terminus) inserted in pBABE-puro vector have been described elsewhere [22], [23]. A pBABE vector containing the cDNA encoding human GLUT1 with an HA epitope tag in its first luminal domain between residues 58 and 59 was constructed by PCR using the oligos 5′-GATCGACTAGGGTCCATAGATACGGAGAATCAATATTACCAGAGATCGATTATCCGTACGATGTTCCTGATTATGCTGAGACCACGCTCACCACGCTCTGG and 3′-GATCGTCGACCTCGAGTCACACTTGGGAATCAGCCCC. The sequence of the PCR parts was verified by sequence analysis (Cogenics/Beckman Coulter).

### Cell Culture

Preadipocytes were cultured and differentiated as described before [23]. Differentiated cultures contained at least 95% adipocytes. To express HA-tagged molecules in adipocytes, preadipocytes were infected with retrovirus as described before [22], except that Plat-E cells were used for the production of virus [24]. L6 myoblasts were infected with retrovirus for the expression of HA-GLUT4 and cultured and differentiated as described previously [22], [25].

Pervanadate was freshly prepared by combining 639 µl PBS, 300 µl 100 mM sodium vanadate and 61 µl 3% H_2_O_2_. After 15 minutes, the pervanadate was used at a concentration of 100 µM [26].

For the induction of insulin resistance, a previously described procedure was used [27], except that adipocytes were incubated for 24 hr with 100 nM insulin.

Human adipocytes were cultured and differentiated as described elsewhere [21].

### Fluorescence-based techniques

The fluorescence-based assay for the detection of cell surface GLUT4 levels and the method to measure GLUT4 internalization have been described previously [22], [28]. In morphological studies, cells were analyzed using a Zeiss LSM 510 confocal laser scanning microscope (Carl Zeiss, Göttingen, Germany) in the C3M Cell Imaging Facility MICA.

### Glucose uptake

Adipocytes, grown in gelatin-coated 12 well plates, were incubated for 2 hours in DMEM with 0.2% BSA and for 5 minutes in KRP (12.5 mM HEPES pH 7.4, 120 mM NaCl, 6 mM KCl, 1.2 mM MgSO_4_, 1 mM CaCl_2_, 0.4 mM NaH_2_PO_4_, 0.6 mM Na_2_HPO_4_). Cells were treated or not with insulin or serum for 20 minutes. To control cells, 50 µM cytochalasin B was added (assay background). Cells were incubated for 3 minutes with 0.1 mM 2-[3H]deoxyglucose (0.28 µCi/well), extensively washed with ice-cold phosphate-buffered saline (PBS), and lysed in 1% Triton X-100. Radioactivity was measured by scintillation counting. To be able to compare serum-induced with insulin-induced 2-DOG uptake, either unlabeled glucose was added to the insulin incubations (Figure 2) or sera were dialyzed (Figure 3) in order to have similar glucose concentrations during the uptake.

### Immunoblotting and immunopurification

3T3-L1 adipocytes were serum-starved for 2 h, incubated for 5 minutes with or without 100 nM insulin or 25% FBS and lysed in ice-cold lysis buffer [29]. For immunopurification (IP), protein A agarose beads (Roche Diagnostics, Meylan, France) were incubated for 1 hr with 5 µg of IRS-1 or IR antibodies at room temperature and for 16 hr with 1 mg of adipocyte lysate at 4°C, subsequently. For control IPs, antibody-bound beads were incubated in the absence of lysate. HRP-conjugated secondary antibodies were visualized using chemiluminescence reagent (Roche Diagnostics) and a CCD camera-based imager (LAS-3000, Fujifilm; St. Quentin en Yvelines, France). Relative intensities were quantitated using MultiGauge software (Fujifilm).

### Column chromatography

Human serum (0.5 ml) was fractionated on a 24 ml Sephacryl 200-HR column using an elution buffer consisting of 150 mM NaCl and 10 mM tris pH 7.4 and a flow rate of 5.0 ml/hr. Calibration markers blue dextran (void volume; 2000 kDa), amylase (200 kDa), alcohol dehydrogenase (150 kDa), bovine serum albumin (67 kDa), ovalbumine (43 kDa), chymotrypsinogen (26 kDa), RNase A (14 kDa) and 2-[3H]deoxyglucose (0.2 kDa) were fractionated using identical volumes and flow rates. Serum fractions (0.75 ml) were concentrated 5 times using 3K Amicon Ultra concentrators before subjecting to the fluorescence-based GLUT4 assay described above.

### Statistics

All data are presented as average ± SD. Experiments were performed at least three times. Representative experiments are shown. Comparisons between data sets were evaluated using two-tailed Student's *t*-tests and comparisons between dose-response curves were evaluated using nonlinear four-parameter sigmoidal dose - response curve fittings and F-tests (Graphpad Prism software). Differences between data sets were considered statistically different when P<0.05.

## Results

### Serum induces the translocation of GLUT4 towards the cell surface

**Figure 1:**
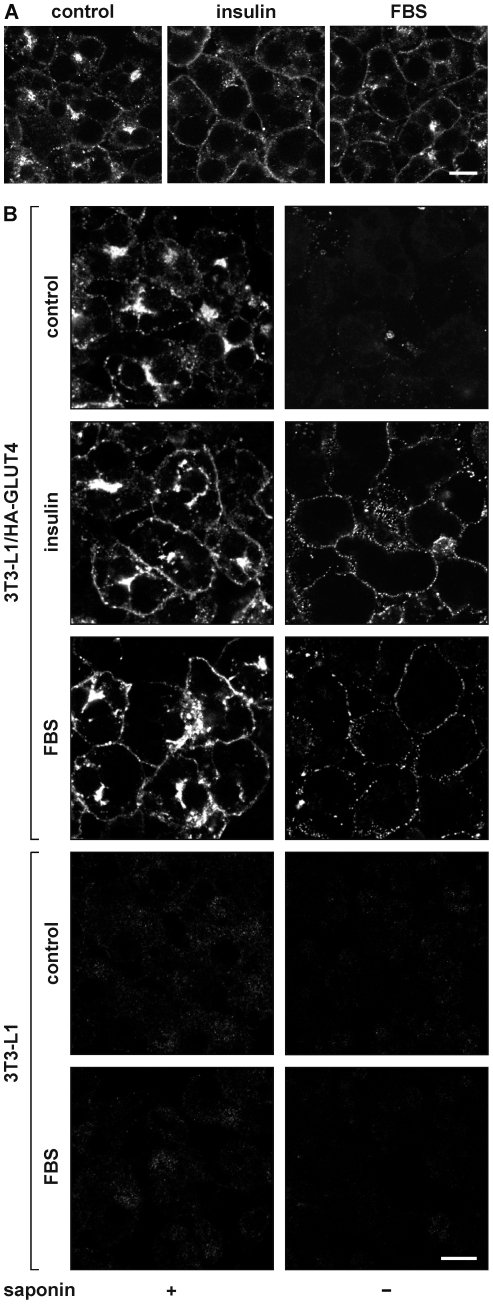
Effect of serum on the intracellular localization of GLUT4. 3T3-L1 adipocytes (***A***) or HA-GLUT4-expressing adipocytes (***B***) were incubated for 20 minutes with 100 nM insulin, 50% FBS, or left untreated (control). Upon fixation, cells were immunolabeled with anti-GLUT4 antibody to label endogenous GLUT4 (*A*) or with anti-HA in the absence (right panels) or presence of saponin (left panels) to label HA-GLUT4 at the cell surface or total cellular HA-GLUT4, respectively (*B*). Control adipocytes that did not express HA-GLUT4 were used to analyse the specificity of the anti-HA labeling (4 lower panels in *B*). Bar, 10 µm.

Insulin is the master regulator of glucose homeostasis via its action on intracellular GLUT4 traffic. To test whether there exist other factors in serum that regulate cell surface GLUT4 levels, 3T3-L1 adipocytes were incubated for 20 minutes with 100 nM insulin, 50% fetal bovine serum (FBS), or left untreated. Cells were subsequently immunolabeled with anti-GLUT4 antibody, and analyzed by microscopy (Figure 1A). We observed that both insulin and serum induced the translocation of endogenous GLUT4 towards the cell surface. As this labeling does not distinguish between GLUT4 molecules localized at the cell surface or just beneath, we expressed ectopic GLUT4 in 3T3-L1 adipocytes bearing an HA-epitope tag within its first extracellular domain. Permeabilized and intact cells were immunolabeled with anti-HA antibody (Figure 1B). The permeabilized adipocytes (left panels) showed that both insulin and FBS increased the appearance of HA-GLUT4 near or at the plasma membrane, while the non-permeabilized cells (right panels) clearly demonstrated an increase in the amounts of GLUT4 at the cell surface upon stimulation with insulin or FBS. The absence of signal in control adipocytes (bottom panels), demonstrated that the immunolabel in FBS-treated HA-GLUT4-expressing adipocytes was specific.

### Quantitative analysis of serum-induced GLUT4 translocation and glucose uptake

**Figure 2:**
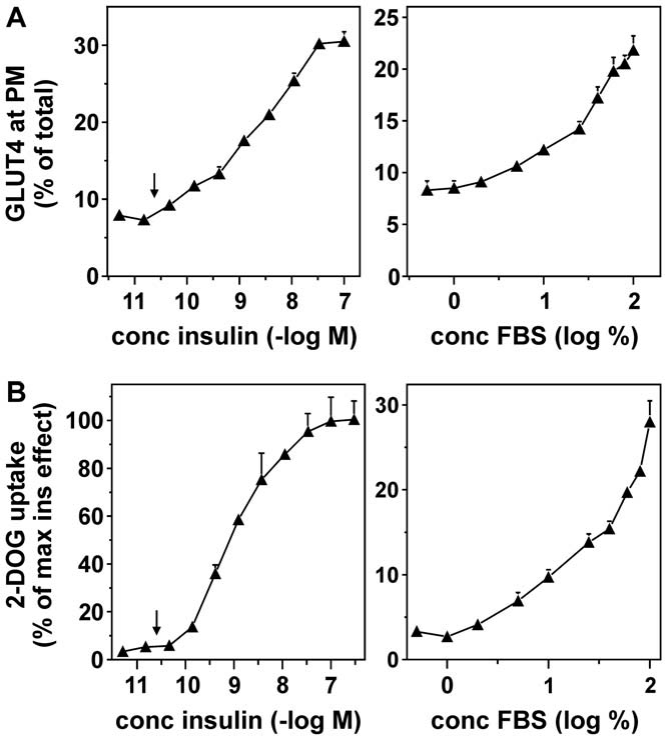
Concentration-dependent GLUT4 translocation and glucose uptake in 3T3-L1 adipocytes in response to insulin and FBS. (***A***) HA-GLUT4-expressing 3T3-L1 adipocytes cultured in 96 well plates were incubated with the indicated concentrations of insulin (left panel) or FBS (right panel), after which the cells were fixed, incubated with or without saponin, and quantitatively immunolabeled for HA signal as described in *Experimental*. The amounts of GLUT4 at the plasma membrane (non-permeabilized cells) was expressed as percentage of total cellular GLUT4 levels (permeabilized cells). (***B***) 3T3-L1 adipocytes were incubated for 20 minutes with the indicated concentrations of insulin (left panel) or FBS (right panel), followed by a three minute incubation with radiolabeled 2-DOG. Cellular 2-DOG uptake was determined for each condition and expressed as percentage of glucose uptake in the presence of saturating concentrations of insulin (100 nM). Arrows in left panels indicate the concentration of insulin in undiluted FBS, demonstrating that the effects of FBS are not mediated by insulin.

Next, we measured GLUT4 translocation quantitatively, using an assay that is based on the culture and labeling of HA-GLUT4-expressing adipocytes in 96 well plates [4]. Cells were processed as described above but fluorescence was analyzed using a fluorescence microplate reader. This revealed that the effect of FBS on cell surface GLUT4 levels was concentration-dependent (Figure 2A). To determine whether the FBS-induced translocation of GLUT4 was accompanied by an increase in glucose transport across the plasma membrane, cellular glucose uptake was measured (Figure 2B). Similarly to its effect on GLUT4 translocation, FBS induced glucose uptake in a concentration-dependent manner. The arrows in the left panels indicate the concentration of insulin in undiluted serum, demonstrating that the observed effects of FBS were not mediated by insulin (see also Table S1). Remarkably, compared with insulin, the increase in cell surface GLUT4 levels in response to FBS was accompanied by a relatively small increase in glucose uptake. This could be due to a smaller effect of FBS on the activity of GLUT4 [30], [31]. Stimulating the cells for various times periods demonstrated that the smaller effect of FBS on glucose transport was independent of the length of stimulation (Figure S1).

### The effect of serum on GLUT4 is not due to inhibition of its internalization and is not mediated by PDGF, IGF-1, or HGF

To establish whether the effect of FBS on GLUT4 was due to an increase in exocytosis or to a reduction in internalization, GLUT4 internalization was measured (Figure S2). In brief, HA-GLUT4-expressing adipocytes were incubated with insulin to increase cell surface GLUT4 levels, cooled down on ice, acid stripped to remove insulin, incubated with anti-HA antibody, and washed to remove non-bound antibody. Upon transfer to 37°C, the cells were incubated with or without 25% FBS to allow the antibody to internalize. Following anti-HA immunolabeling, the cells were scored for the presence of internalized label. Unexpectedly, we observed that FBS increased the GLUT4 internalization rate. Therefore, the positive effect of FBS on cell surface GLUT4 levels must be due to an increased exocytosis rate. Control adipocytes that were incubated with 0.45 M sucrose displayed a large reduction in internalization rate [32].

To determine whether the GLUT4-translocating activity of serum was due to the presence of factors that have previously been shown to alter cell surface GLUT4 levels or glucose transport under certain conditions, we tested platelet-derived growth factor (PDGF; [33]), insulin-like growth factor-1 (IGF-1; [34]), and hepatocyte growth factor (HGF; [35]) in our cell system (Figure S3). None of these factors increased cell surface GLUT4 levels (Figure S3A), though all factors activated signal transduction pathways (Figure S3B).

### The effect of serum on GLUT4 is independent of serum origin and cell type

**Figure 3:**
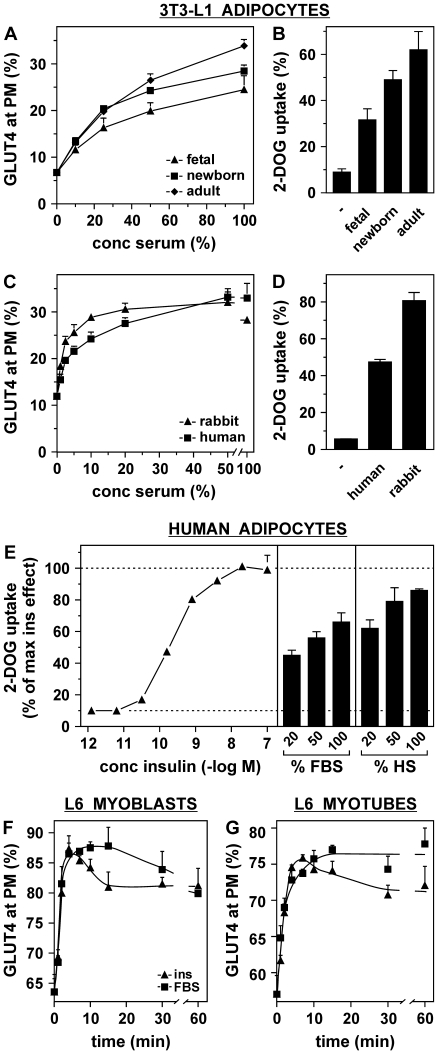
The effect of serum on GLUT4 is independent of serum origin and cell type. (***A***) 3T3-L1 adipocytes were incubated for 20 minutes with the indicated concentrations of fetal, newborn, and adult bovine serum and cell surface GLUT4 levels were determined. (***B***) 3T3-L1 adipocytes were incubated for 20 minutes with 100 nM insulin, 50% serum or left untreated and cellular glucose uptake was measured and expressed as percentage of insulin-stimulated glucose uptake. (***C***, ***D***) Rabbit and human sera were analyzed as under (*A*) and (*B*). (***E***) Human adipocytes were incubated for 20 minutes with various concentrations of insulin, FBS, or human serum (HS) and glucose uptake was measured and expressed as percentage of maximal glucose uptake in response to insulin. (***F***, ***G***) HA-GLUT4-expressing myoblasts (*F*) and myotubes (*G*) were stimulated with either 100 nM insulin or 25% FBS and cell surface GLUT4 levels were determined. Serum insulin concentrations are depicted in Table S1.

To determine whether the translocation-inducing serum factor was exclusively present in FBS, we also studied newborn bovine, adult bovine, rabbit and human serum (Figure 3A–D). We observed that in 3T3-L1 adipocytes, all tested sera induced GLUT4 translocation and glucose uptake that could not be accounted for by serum insulin levels (Table S1).

To test whether the action of serum on GLUT4 was dependent on cell type, human adipocytes, rat myoblasts, and rat myotubes were investigated. In *in vitro* differentiated human adipocytes, FBS and human serum induced an insulin-independent increase in glucose uptake (Figure 3E). In L6 myoblasts and myotubes, both insulin and FBS acutely increased the amount of HA-GLUT4 at the plasma membrane (Figure 3F and G). For both muscle cell models, the ED50 values for insulin and FBS were around 5 nM and 1%, respectively (data not shown). This indicated that, compared with 3T3-L1 adipocytes, these cells were even more sensitive to FBS and that also in these cells FBS induced GLUT4 translocation independent of insulin.

### Serum augments also the cell surface levels of GLUT1 and the transferrin receptor (TfR), but only for GLUT4 the increase is additive to the effect of insulin

**Figure 4:**
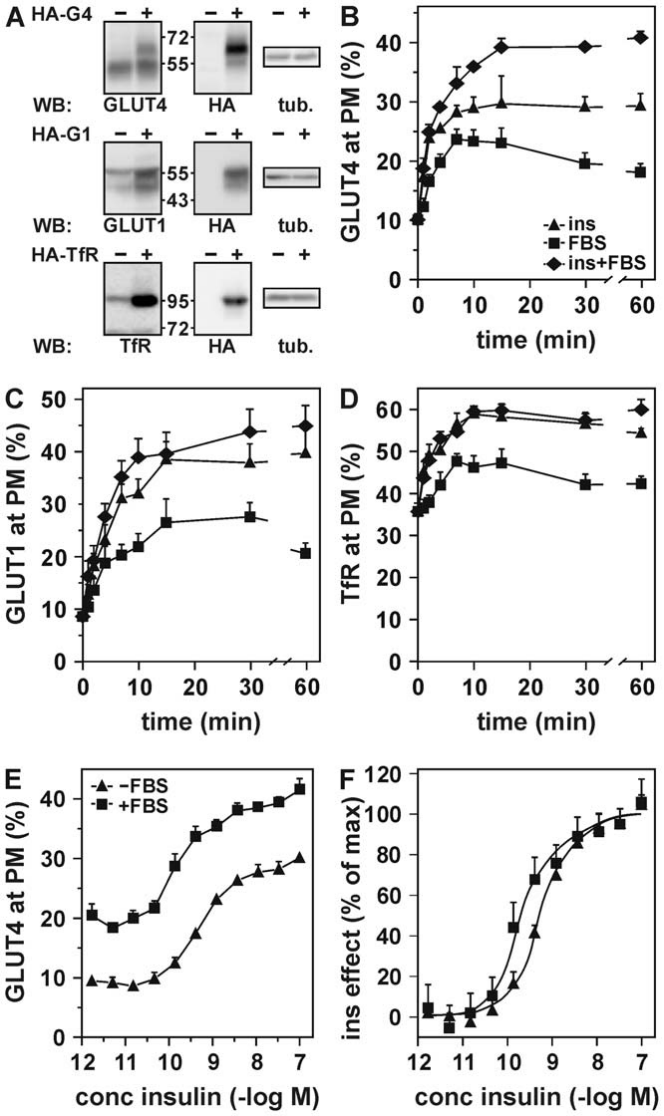
The effect of FBS is additive to that of insulin and is not limited to GLUT4. (***A***) Lysates of 3T3-L1 adipocytes expressing HA-GLUT4, HA-GLUT1, or HA-TfR, and control adipocytes were subjected to SDS-PAGE and immunoblotted using the indicated antibodies. 3T3-L1 adipocytes expressing HA-GLUT4 (***B***), HA-GLUT1 (***C***), or HA-TfR (***D***) were stimulated for various times with 100 nM insulin, 25% FBS or both and relative cell surface GLUT4 levels were determined. (***E***) Adipocytes were incubated for 20 minutes with the indicated concentrations of insulin in the absence or presence of 25% FBS, and relative cell surface GLUT4 levels were determined. (***F***) Data from (*E*) were transformed in dose-response curves in which the effects of the various insulin concentrations were related to the maximal insulin effects (difference between 0 and 100 nM insulin) in the absence and presence of FBS. EC50 of insulin in absence and presence of FBS is 0.57 and 0.19 nM insulin, respectively. P<0.0001.

As insulin also increases the amount of GLUT1 and TfR at the plasma membrane, we investigated whether serum would have a similar effect. Therefore, we analyzed the kinetics of the appearance of GLUT4, GLUT1, and TfR at the cell surface in response to insulin, FBS, or both (Figure 4). As for GLUT4, GLUT1 and TfR proteins were studied that contained an HA epitope tag within their extracellular domain. In particular for GLUT4 and GLUT1, overexpression levels of these HA-tagged molecules were moderate (Figure 4A). Colocalization studies with syntaxin 13 showed that the intracellular localization of HA-tagged GLUT1 did not differ from that of endogenous GLUT1 (Figure S4). While insulin-increased cell surface GLUT4 levels were relatively stable, FBS led to a somewhat transient increase in GLUT4 at the plasma membrane, reaching maximal levels after 7–10 minutes (Figure 4B). Remarkably, the effects of insulin and FBS were largely additive and moreover, the additive effect of FBS did not decline throughout the duration of the experiment. The effect of FBS was not specific to GLUT4 as GLUT1 and TfR levels were also increased at the plasma membrane upon FBS stimulation (Figure 4C and D). An effect of serum on GLUT1 has been demonstrated before [36]. While GLUT1 and TfR were also sensitive to insulin stimulation, in accordance with previous studies [5], the effects of FBS and insulin were not additive. Treatment of HA-GLUT4-expressing adipocytes with various concentrations of insulin in the absence and presence of FBS demonstrated that along the entire insulin concentration range, FBS increased cell surface GLUT4 levels (Figure 4E). These data suggest that the insulin and FBS signaling pathways leading to increases in cell surface GLUT4 levels are largely distinct. Moreover, FBS sensitized the adipocytes to insulin, in that the insulin dose-response curve shifted leftwards concomitant with a significant reduction in EC50 of 0.57 nM to 0.19 nM (Figure 4F).

### Investigation of the additive effects of serum and insulin demonstrates important differences in signaling

**Figure 5:**
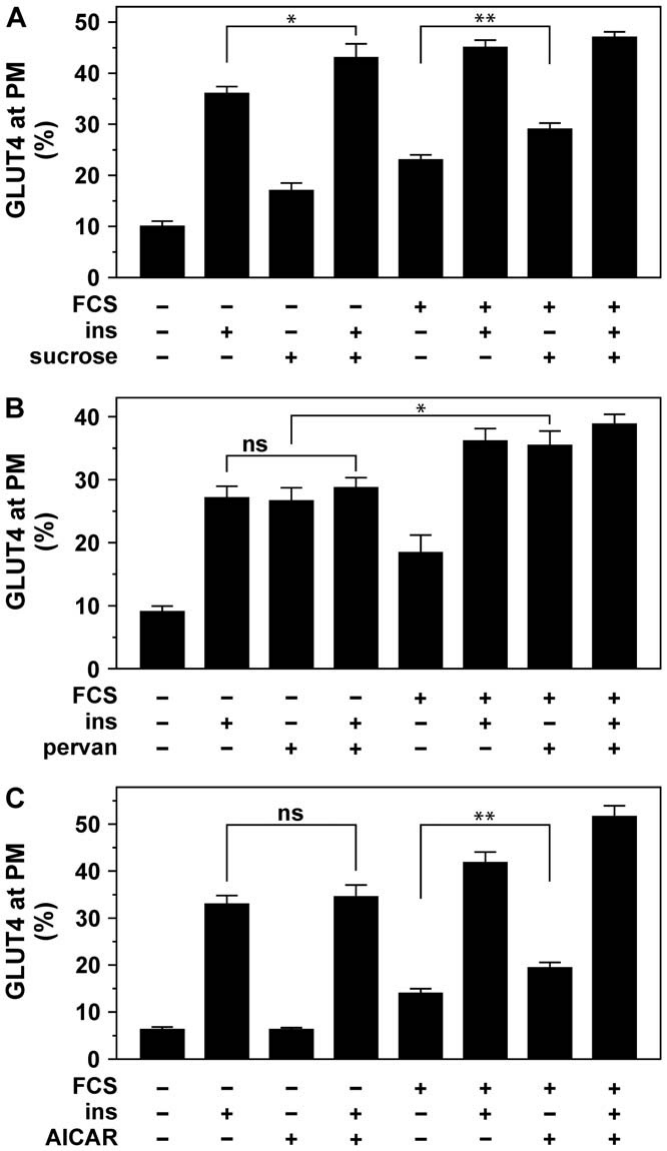
The additive effects of FBS and insulin on various stimuli are distinct. (***A***) Adipocytes were incubated for 1 hour in the absence or presence of 0.45 M sucrose, following a 10 minute incubation with 100 nM insulin, 25% FBS or control medium. Cell surface GLUT4 levels were determined. (***B***) Cells were incubated with or without 100 µM pervanadate for a total of 20 minutes. Insulin (100 nM) and FBS (25%) were added for the final 10 minutes. Amounts of GLUT4 at the plasma membrane were determined (**C**) Cells were incubated for 40 minutes with or without 5 mM AICAR, 100 nM insulin, and 25% FBS, upon which relative cell surface GLUT4 levels were established. *P<0.001; **P<0.0001; ns, non-significant.

In order to obtain evidence for differences in insulin- and serum-induced signaling leading to the translocation of GLUT4, we analyzed their effect on cell surface GLUT4 levels in cells exposed to hyperosmolarity, protein tyrosine phosphatase inhibitor pervanadate, and AMPK activator AICAR (Figure 5). These three conditions have been shown to increase cell surface GLUT4 levels in 3T3-L1 adipocytes, L6 myoblasts, and/or L6 myotubes [26], [37], [38]. Hyperosmolarity (0.45 M sucrose; [32]) modestly but significantly increased cell surface GLUT4 levels in the absence and presence of insulin and FBS (Figure 5A). While the pervanadate-induced increase in cell surface GLUT4 levels could not be further enhanced by insulin, the effects of pervanadate and FBS were partially additive (Figure 5B). AICAR did not increase the amount of GLUT4 at the cell surface in unstimulated and insulin-stimulated cells (Figure 5C). However, the effect of FBS on GLUT4, in the absence as well in the presence of insulin, was significantly enhanced by AICAR. This confirmed that the signaling pathways that are activated by insulin and FBS and lead to GLUT4 translocation are likely to be distinct.

### The serum factor that induces GLUT4 translocation activates signaling pathways that are distinct from those activated by insulin

**Figure 6:**
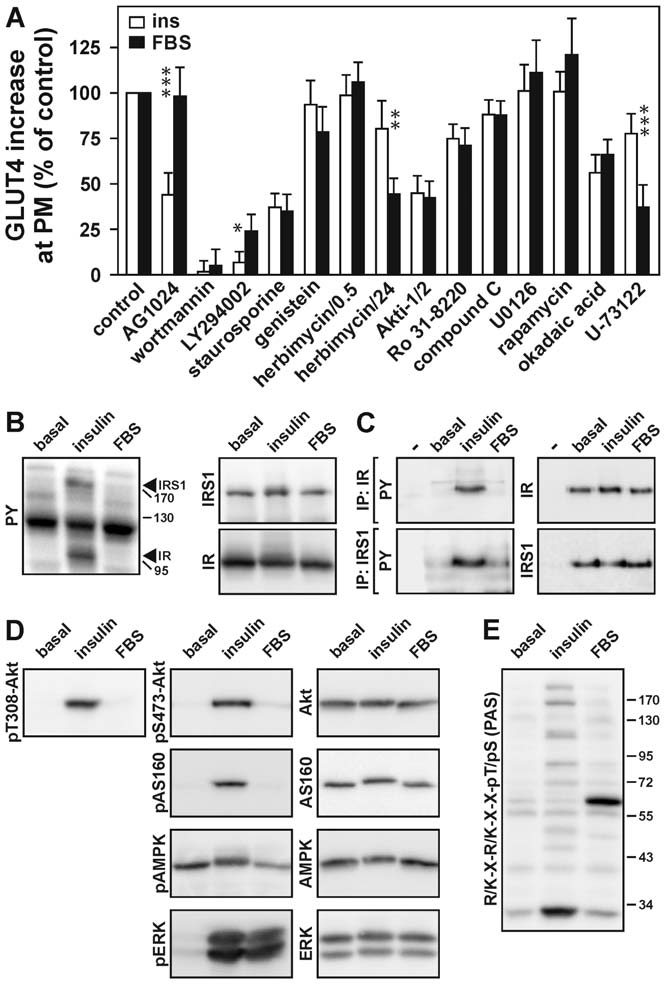
The intracellular signal transduction pathways involved in FBS-induced GLUT4 translocation are partially different from those of insulin. (***A***) Adipocytes were preincubated for 30 minutes with 25 µM AG1024, 100 nM wortmannin, 50 µM LY294002, 30 µM staurosporine, 300 µM genistein, 2 µM herbimycin A (‘herbimycin/0.5’), 10 µM Akti-1/2, 10 µM Ro 31-8220, 40 µM compound C, 10 µM U0126, 40 nM rapamycin, 1 µM okadaic acid, or 10 µM U-73122, or for 24 hours with 1.75 µM herbimycin A (‘herbimycin/24’). Insulin (100 nM) or FBS (50%) was added, cells were incubated for a further 10 minutes, and cell surface GLUT4 levels were determined. To demonstrate the effect of the inhibitors, the relative increase in cell surface GLUT4 levels in the presence of inhibitors was expressed as percentage of the increase in the absence of inhibitors. (***B–E***) Adipocytes were stimulated for 5 minutes with 100 nM insulin, 25% FBS or left untreated. Lysates were either directly subjected to SDS-PAGE and immunoblotting using the indicated antibodies (*B*,*D*,*E*), or first subjected to immunopurification (IP), in the case of the insulin receptor (IR) and IRS-1 (*C*). *P<0.05; **P<0.01; ***P<0.005.

We next sought further proof that the two signaling pathways are indeed distinct. To this aim, we applied two approaches. In the first approach, we investigated insulin- and serum-induced GLUT4 translocation in the presence of inhibitors of various signaling molecules that are part of the insulin signal transduction cascade (Figure 6A). These compounds inhibit insulin receptor tyrosine kinase activity (AG1024; [39], PI 3-kinase (wortmannin and LY294002; [40]), serine/threonine kinases (staurosporine; [41]), tyrosine kinases (genistein and herbimycin A; [42]), Akt (Akti-1/2; [43]), PKC (Ro 31-8220; [44]), AMPK (compound C; [45]), MEK (U0126; [21]), mTOR (rapamycin; [46]), protein phosphatases 1 and 2A (okadaic acid; [2]), and phospholipase C (U-73122; [47]). This analysis demonstrated that while serum- and insulin-induced GLUT4 translocation were similarly affected by most of these inhibitors, there were three important differences. Tyrphostin AG1024 reduced insulin-induced GLUT4 translocation for more than 50% while it left serum action unaffected. A prolonged exposure of the adipocytes to herbimycin A, a condition that is associated with a reduction in expression and signaling by the insulin receptor in MCF-7 cells [48], decreased serum-induced GLUT4 translocation to a greater extent than translocation induced by insulin. A larger effect on serum action was also found for phospholipase C inhibitor U-73122 [47].

In a second approach, we used Western blot analyses to study the effect of FBS on several signaling molecules that are known to be implicated in GLUT4 translocation (Figure 6B–E). In contrast to insulin, FBS did not induce tyrosine phosphorylation of the insulin receptor and IRS-1 (Figure 6B and C) nor threonine and serine phosphorylation of Akt and its downstream effector AS160 (Figure 6D). We did observe that FBS largely increased phosphorylation of MAP kinases ERK1 and ERK2. However, these MAP kinases do not play a role in GLUT4 translocation [49]. Finally, we analyzed the presence of phosphorylated Akt substrates using an antibody directed against a Phosphorylated Akt Substrate consensus sequence (PAS antibody; Figure 6E). Several proteins displayed immunoreactivity in insulin-stimulated cells. These proteins were not phosphorylated in FBS-treated cells. This is in accordance with the absence of phosphorylation of Akt substrate AS160. However, in these cells there was one particular protein of approximately 60 kDa that was largely phosphorylated in response to FBS. This protein did not appear to be phosphorylated upon insulin stimulation, making it tempting to speculate that this protein and its upstream kinase may be involved in FBS-induced GLUT4 translocation. These data indicate that while the serum-activated signaling pathways that lead to GLUT4 translocation remain to be revealed, it is clear that they are, at least in part, distinct from those involved in insulin signaling.

### Serum-induced GLUT4 translocation is maintained in insulin resistance

So far, our results suggested that the effect of the serum factor on GLUT4 might be maintained in insulin resistance, as the action of FBS on GLUT4 was largely additive to that of insulin (Figure 4) while distinct signaling pathways appeared to be involved (Figure 5 and 6).

**Figure 7:**
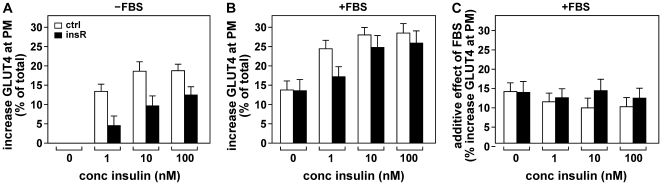
The effect of FBS on GLUT4 persists in insulin-resistant adipocytes. 3T3-L1 adipocytes rendered insulin-resistant by a 24 hour 100 nM insulin treatment (black bars, ‘insR’) or control adipocytes (white bars, ‘ctrl’) were incubated for 20 minutes with the indicated concentrations of insulin in the absence (***A***) or presence of 25% FBS (***B***) and increases in cell surface GLUT4 levels were determined. (***C***) Values of (*A*) were subtracted from those of (*B*) to calculate the additive effect of FBS. In (*A*), control and insulin-resistant cells were significantly different for all three insulin concentrations (P<0.005), while in (*C*) there were no significant differences between the cells.

To investigate this, insulin resistant 3T3-L1 adipocytes were incubated with different concentrations of insulin in the absence and presence of 25% FBS and cell surface GLUT4 levels were measured (Figure 7). As expected, insulin resistant cells displayed reduced GLUT4 translocation in response to insulin (panel A). Notably, the presence of FBS during the insulin incubation largely increased cell surface GLUT4 levels in both insulin-sensitive and insulin-resistant cells (panel B). Calculation of the additive effect of FBS demonstrated that its action on GLUT4 persisted in insulin-resistant cells (panel C). These data demonstrate that the effect of the serum factor is not only independent of insulin but also that its effect is fully preserved in insulin resistance.

### The GLUT4-translocating activity of serum fractionates in two peaks in size exclusion chromatography

**Figure 8:**
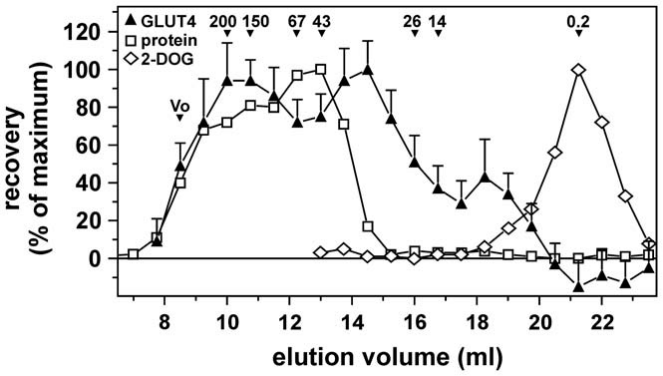
Size exclusion chromatography of serum reveals that multiple proteins or protein complexes are involved in serum-induced GLUT4 translocation. Human serum to which a trace amount of 2-[3H]deoxyglucose was added was fractionated as described in *Experimental*. Fractions were evaluated for their effect on cell surface GLUT4 levels. The maximum increase was set to 100%. Protein concentrations and 2-DOG were also expressed as percentage of maximum. The void volume (Vo) and the elution profile of the calibration standards (200-14 kDa) are indicated. The (0.2 kDa) 2-DOG peak marks the end of the elution process.

To investigate the nature of the putative factor in serum, we fractionated human serum on a Sephacryl 200HR column and analyzed the effect of the fractions on cell surface GLUT4 levels (Figure 8). This revealed two major peaks estimated at ∼170 kDa and ∼33 kDa and a minor peak at around 7 kDa, suggesting that either at least two independent factors contribute to the GLUT4-translocating activity in serum or that part of the implicated factor exists in a protein complex.

## Discussion

Here, we present evidence for the existence of GLUT4-translocating activity in serum and show that the responsible factor is not insulin, IGF-1, PDGF, or HGF. Moreover, neither the insulin receptor nor IRS-1 are tyrosine phosphorylated upon serum stimulation. Also, insulin- but not serum-induced GLUT4 translocation is inhibited by tyrphostin AG1024, known to target the IGF-1 receptor and, to a somewhat lesser extent, the insulin receptor [39]. This further confirms that the serum effect is not mediated by insulin or IGF-1. This is important as an insulin-like activity of serum on adipocytes has previously been attributed to NSILA [50], later identified as IGF [51]. We have found insulin-independent GLUT4-translocating activity in all sera that we have tested (i.e. fetal, newborn and adult bovine serum, human serum and rabbit serum). Our studies have demonstrated that multiple adipocyte and muscle cell models display serum-induced GLUT4 translocation. Taken together, this demonstrates that the factor is generally present in serum and acts on all cell types that are implicated in postprandial glucose uptake. Importantly, our data imply a novel signal transduction pathway leading to GLUT4 translocation and glucose uptake, which is preserved in insulin resistance.

Several of our observations demonstrate that the mode of action of FBS is largely different from that of insulin. First, while insulin action regarding GLUT4 was largely restricted in insulin-resistant adipocytes, serum-induced GLUT4 translocation was fully preserved. Second, the effect of FBS is largely additive to that of insulin. Third, while the effect of pervanadate on GLUT4 is partially additive to that of FBS, it is not additive to the effect of insulin. Fourth, incubation of the adipocytes with the AMP kinase activator AICAR increases cell surface GLUT4 levels when added together with FBS but not with insulin. Fifth, our inhibitor studies demonstrated that the FBS effect was much more sensitive to long-term herbimycin A treatment and phospholipase C inhibitor U-73122 than that of insulin. Finally, analyses of the insulin signaling pathway demonstrated that the downstream signaling molecules involved in insulin-induced GLUT4 translocation are not activated by FBS.

Our data demonstrate that there exists an as yet unidentified signaling pathway that leads to GLUT4 recruitment and glucose uptake and that this pathway is activated by a serum factor. Here, we have shown that this pathway does not imply AMP kinase, as serum-induced GLUT4 translocation was not inhibited by AMPK inhibitor compound C. Moreover, the translocation of GLUT4 in response to FBS was enhanced by AMPK activator AICAR, while FBS itself did not alter phosphorylation of AMPK or its downstream effector acetyl CoA carboxylase (ACC; not shown). The effect of FBS on GLUT4 was inhibited more than 50% by inhibitors of PI 3-kinase as well as by staurosporine, 24 hour herbimycin A treatment, Akti-1/2, and U73122, indicating the involvement of PI 3-kinase, a serine/threonine protein kinase, and phospholipase C. Although serum-induced GLUT4 translocation was inhibited by Akti-1/2, serum did not induce phosphorylation of Akt2 nor of its downstream effector AS160 [52], suggesting that the inhibition by Akti-1/2 may be due to the involvement of a structurally similar serine/threonine kinase whose activity is reduced by Akti-1/2. In accordance with this possibility, serum but not insulin induced the phosphorylation of a ∼60 kDa protein that was reactive with the PAS antibody, raised against a phosphorylated Akt substrate consensus sequence. These results suggest that another member of the AGC kinase family may be involved in the serum action as many members of this family share the same substrate phosphorylation sequence.

At present it is unclear what is the nature of the serum factor(s). The factor is likely to be a protein as the GLUT4-translocating activity is collected in the protein fraction upon ammonium sulfate precipitation (data not shown). Moreover, on a size exclusion column, it fractionates into two major peaks (estimated M_r_ 33 and 170 kDa), while it is not retained on a hydrophobic LH20 column (not shown). This may imply that two proteins are involved. Alternatively, the implicated factor may in part be associated with other proteins. Interestingly, heating FBS for up to 10 minutes at 100°C precipitates 75% of the protein content, while leaving the serum GLUT4-translocating activity intact (not shown), implying thermostability and suggesting that it is not the mere presence of a bulk of protein that induces GLUT4 translocation but that the effect is more specific. This is also evident from our size exclusion chromatography studies, in which GLUT4-translocating activity is present in fractions that contain minimal amounts of protein.

This factor may be regulated in a way similar to insulin in that its serum levels may oscillate depending on food intake. Moreover, its serum levels may be altered in insulin resistance *in vivo*. To date, we have not yet investigated these issues. The 96 well plate technique that has been used throughout this study will largely aid in addressing these questions and will likely lead to its purification and identification, and to the definition of its (patho)physiological relevance. Unfortunately, the activity-based purification of unidentified proteins from serum has proven often to be a difficult and laborious task.

Taken together, we have demonstrated the presence of a factor (or several factors) in serum that induce GLUT4 translocation in a manner that is largely independent of insulin and insulin signaling and whose effects are fully maintained in insulin resistance. Hence, this factor may prove to have beneficial effects in type 2 diabetes. Now, the challenging task is to identify this factor and to examine its effects on GLUT4 and glucose uptake under normal conditions and conditions of insulin resistance *in vitro* and *in vivo*.

## Supporting Information

Table S1 **Insulin concentrations in the sera used in the described studies.**
(TIF)

Figure S1 **The relative effect of serum on cellular glucose uptake is lower than its effect on cell surface GLUT4 levels.** FBS was extensively dialyzed against KRP buffer (cut-off 3 kDa). 3T3-L1 adipocytes expressing HA-GLUT4 (***A***) or not expressing HA-GLUT4 (***B***) were serum starved for 2 h in DMEM containing 0.2% BSA, washed with KRP, and incubated for the indicated time periods in the absence (open triangles) or presence of 100 nM insulin (open squares), 25% FBS (filled triangles), 50% FBS (filled squares), or 100% FBS (filled diamonds) in KRP. Subsequently, (***A***) non-permeabilized fixed cells were immunolabeled with anti-HA and fluorescent secondary antibodies followed by measurement of the fluorescence (arbitrary units) or (***B***) the uptake of radiolabeled 2DOG during a 2 min incubation was measured. The dashed line in the panels represents basal cell surface GLUT4 levels/2DOG uptake.
(TIF)

Figure S2 **Serum does not reduce GLUT4 internalization.** 3T3-L1 adipocytes expressing HA-GLUT4 were incubated for 20 min with 100 nM insulin, followed by cooling down on ice, removal of insulin, and cell surface labeling of the cells with anti-HA antibody. Excess antibody was removed, cells were transferred to 37°C, and fixed after various time periods. Permeabilized cells were immunolabeled with fluorescent goat-anti-mouse antibody and analyzed by fluorescence microscopy. Cells were scored for the presence of internalized anti-HA label. Hyperosmolarity (0.45 M sucrose) was included as positive control.
(TIF)

Figure S3 **The effect of FBS on GLUT4 is not mediated by PDGF, IGF-1, or HGF.** (***A***) Adipocytes were incubated for 20 minutes with various concentrations of the indicated ligands and relative cell surface GLUT4 levels were determined. (***B***) 3T3-L1 adipocytes and preadipocytes were incubated for 5 minutes with 100 nM insulin, 2 nM PDGF-BB, 25 nM IGF-1, or 2.6 nM HGF, and lysate samples were subjected to SDS-PAGE and immunoblotting using phospho-ERK and ERK antibodies.
(TIF)

Figure S4 **HA-tagged GLUT1 is correctly localized.** 3T3-L1 adipocytes expressing GLUT1 with an HA-epitope tag in its first extracellular domain were immunolabeled using anti-HA and anti-syntaxin 13 antibodies. Control adipocytes were immunolabeled using anti-GLUT1 and anti-syntaxin 13 antibodies. Note that the localization of HA-GLUT1 is similar compared with endogenous GLUT1. Bar, 5 µm.
(TIF)
