# Supplementary figures and images for: A Serum Factor Induces Insulin-Independent Translocation of GLUT4 to the Cell Surface which Is Maintained in Insulin Resistance
Source: PLoS One. 2010 Dec 20;5(12):e15560. doi: 10.1371/journal.pone.0015560 (PMC3004919; doi:10.1371/journal.pone.0015560)

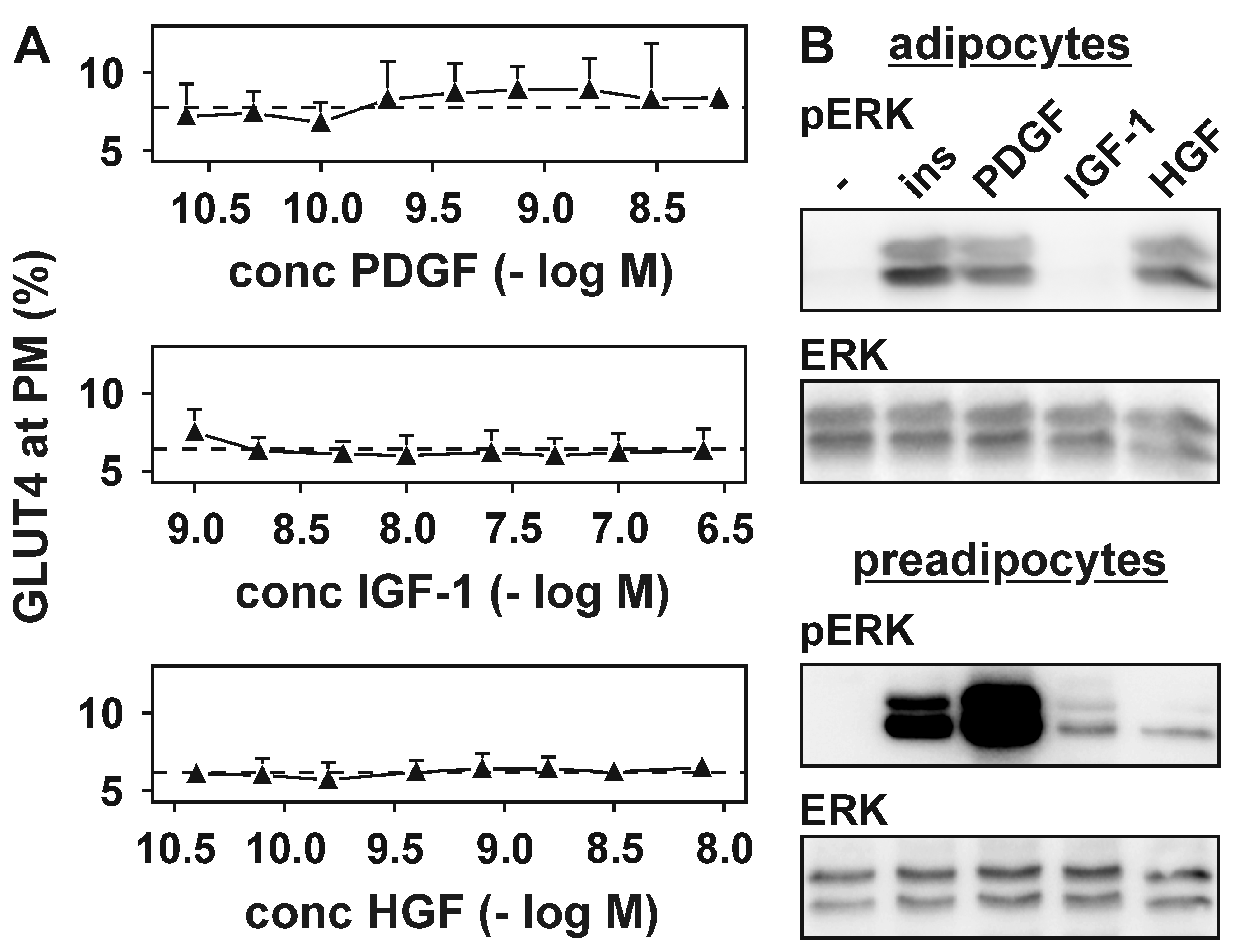

Supplement: Figure S3 — The effect of FBS on GLUT4 is not mediated by PDGF, IGF-1, or HGF. (A) Adipocytes were incubated for 20 minutes with various concentrations of the indicated ligands and relative cell surface GLUT4 levels were determined. (B) 3T3-L1 adipocytes and preadipocytes were incubated for 5 minutes with 100 nM insulin, 2 nM PDGF-BB, 25 nM IGF-1, or 2.6 nM HGF, and lysate samples were subjected to SDS-PAGE and immunoblotting using phospho-ERK and ERK antibodies. (TIF) [file pone.0015560.s004.tif]

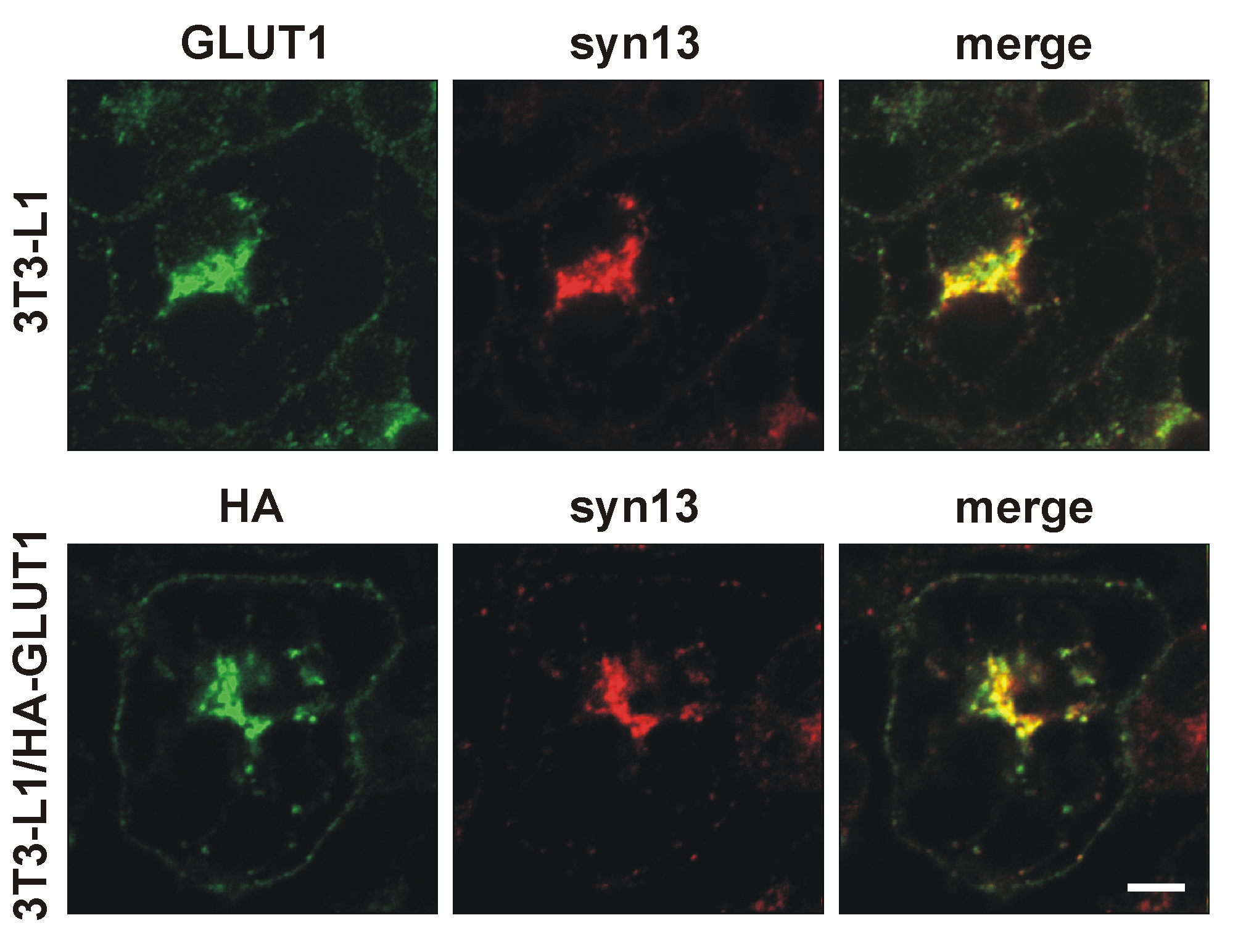

Supplement: Figure S4 — HA-tagged GLUT1 is correctly localized. 3T3-L1 adipocytes expressing GLUT1 with an HA-epitope tag in its first extracellular domain were immunolabeled using anti-HA and anti-syntaxin 13 antibodies. Control adipocytes were immunolabeled using anti-GLUT1 and anti-syntaxin 13 antibodies. Note that the localization of HA-GLUT1 is similar compared with endogenous GLUT1. Bar, 5 µm. (TIF) [file pone.0015560.s005.tif]
